# The Roles of Post-translational Modifications in the Context of Protein Interaction Networks

**DOI:** 10.1371/journal.pcbi.1004049

**Published:** 2015-02-18

**Authors:** Guangyou Duan, Dirk Walther

**Affiliations:** Max Planck Institute for Molecular Plant Physiology, Potsdam-Golm, Germany; Indiana University, UNITED STATES

## Abstract

Among other effects, post-translational modifications (PTMs) have been shown to exert their function via the modulation of protein-protein interactions. For twelve different main PTM-types and associated subtypes and across 9 diverse species, we investigated whether particular PTM-types are associated with proteins with specific and possibly “strategic” placements in the network of all protein interactions by determining informative network-theoretic properties. Proteins undergoing a PTM were observed to engage in more interactions and positioned in more central locations than non-PTM proteins. Among the twelve considered PTM-types, phosphorylated proteins were identified most consistently as being situated in central network locations and with the broadest interaction spectrum to proteins carrying other PTM-types, while glycosylated proteins are preferentially located at the network periphery. For the human interactome, proteins undergoing sumoylation or proteolytic cleavage were found with the most characteristic network properties. PTM-type-specific protein interaction network (PIN) properties can be rationalized with regard to the function of the respective PTM-carrying proteins. For example, glycosylation sites were found enriched in proteins with plasma membrane localizations and transporter or receptor activity, which generally have fewer interacting partners. The involvement in disease processes of human proteins undergoing PTMs was also found associated with characteristic PIN properties. By integrating global protein interaction networks and specific PTMs, our study offers a novel approach to unraveling the role of PTMs in cellular processes.

## Author Summary

The function of proteins is frequently modulated by chemical modifications introduced after translation from RNA. These post-translational modifications (PTMs) have been shown to also influence the interaction between proteins carrying them. We tested whether specific PTM-types characterized by attaching different chemical groups are associated with proteins with characteristic and possibly strategic positions within the network of all protein interactions in cellular systems. Based on network-theoretic analyses of PTMs in the context of protein interaction networks of nine selected species, we indeed observed distinctive properties of twelve PTM-types tested. Phosphorylation was found associated with proteins in central locations with the broadest interaction scope, while glycosylation was more prominent in proteins at the periphery of the web of all protein interactions. The involvement in disease processes of human proteins undergoing PTMs was also found associated with characteristic protein interaction network properties. Our study highlights common and specific roles of the various PTM types in the orchestration of molecular interactions in cells.

## Introduction

As chief actors within living cells, proteins serve diverse functions such as catalysis, transport, structural building material and many others [1]. While the human gene set was estimated at about 25,000 genes [2], the human proteome size is expected to be far larger and estimated at over 1 million proteins [3]. Beyond alternative splicing of mRNA as a source of protein diversity, post-translational modifications (PTMs) of proteins further modulate and extend the range of possible protein functions by covalently attaching small chemical moieties to selected amino acid residues. More than 200 different types of PTMs have been identified that affect many aspects of cellular functionalities, such as metabolism, signal transduction, and protein stability [4, 5]. These modifications include phosphorylation, glycosylation, methylation, acetylation, amidation and many other types, see http://www.uniprot.org/docs/ptmlist for a more detailed controlled vocabulary of PTMs curated by UniProt [6]. With technological advances, PTMs can be detected at an ever increasing breadth, precision, and quantity e.g. by using mass spectrometry (MS) based methods [7]. Several databases have been established to store the obtained information, such as UniProt [8], dbPTM [9], PTMCuration [10], PTMcode [11] and many others. Among them, a number species-specific databases have been developed [12–14] offering the opportunity to investigate PTMs in an evolutionary context as well.

Many studies on PTMs have focused on specific types and their relevance for protein function with phosphorylation representing the most actively researched PTM-type [15–19]. More recently, the interplay between different PTM-types has moved into the focus of attention [20–23]. For example, evidence of an interdependence of phosphorylation and acetylation was reported for a genome-reduced bacterium *Mycoplasma pneumoniae* [24]. Furthermore, so-called integrative PTM spots (PTMi) have been identified as site in proteins at which different PTMs operated in a combinatorial manner to modulate protein function [25]. A more global view of the interplay between PTM-types was presented in a study on the co-evolution between 13 frequent PTM types in 8 eukaryotic species [26]. Carboxylation was identified as evolutionarily most conserved, whereas phosphorylation was found among those PTM-types playing a central role in the modulation of the dynamics of protein function. For a recent review on the evolution and functional cross-talk between PTMs, see [27].

In addition to PTMs, protein function is also regulated and mediated by non-covalent protein-protein interactions [28–33]. As many PTMs modulate the binding affinities between proteins by changing the electrostatic or structural properties of the involved interaction sites [29], PTMs and protein-protein interactions are frequently functionally connected. Based on data from the dbPTM database of protein post-translational modifications [9], more than 60% of PTM sites are related to those protein functional domains that were shown to preferentially engage in direct protein-protein interactions suggesting a central regulatory role of PTMs in the modulation of protein interactions, and thus, function.

Therefore, it appears plausible that proteins carrying a particular PTM-type may possess specific interaction characteristics. Indeed, for the important and intensively investigated PTM-type phosphorylation, it was found that in yeast, phospho-proteins engage in many more protein-protein interaction than proteins without phosphorylation sites [34], found similarly in *Arabidopsis thaliana* [35]. Thus, phosphorylation of a single protein potentially leads to a modulation of many different interactions, and thus, molecular processes, simultaneously.

As the different PTMs modify proteins in specific ways, it appears furthermore likely that their consequences on protein-protein interactions may be different as well. This hypothesis formed the starting point for the present study. Specifically, we asked whether different PTM-types are associated with characteristic protein-protein network properties, such as interaction degree, clustering coefficient, and closeness centrality, for those proteins carrying them. We selected these three properties as they each reflect on potential functional role such as scope of impact (degree), diversity of responses (clustering coefficient), and placement within a possible signaling cascade (closeness centrality). Furthermore, we investigated whether those characteristics are conserved across different species, and if the particular functions the different PTMs fulfil may become apparent when inspected from the viewpoint of protein-protein interactions.

## Results

To base our analyses on high-confidence PTM-instances, we restricted our analyses to PTM-sites that have been identified experimentally leaving out all annotated PTMs that are based on computational predictions alone. PTM-site information was collected from 11 different database resources and consolidated into a single set via sequence position information. Currently available datasets proved sufficiently large to conduct statistical analyses on the role of PTMs in the context of protein interaction networks for the following twelve PTM-types: acetylation, amidation, carboxylation, disulfide bond, glycosylation, hydroxylation, methylation, nitrosylation, phosphorylation, proteolytic cleavage, sumoylation, and ubiquitination associated with a set of nine diverse eukaryotic species covering several lineages and kingdoms: mammals (*Homo sapiens*, *Mus musculus*, *Rattus norvegicus*, *Bos taurus*), an invertebrate (*Caenorhabditis elegans*), the insect *Drosophila melanogaster*, fungi (*Saccharomyces cerevisiae*, *Schizosaccharomyces pombe*), and the plant *Arabidopsis thaliana* (Table 1). A*cetylation* is characterized by the attachment of an acetyl group either to the N-terminus of protein or to lysine residues. A*midation* leads to the addition of amide groups to the C-terminus of proteins. During *carboxylation*, a carboxylic group is added to glutamate residues. *Glycosylation* includes all O-linked (serine, threonine, tyrosine residues) or N-linked (arginine and asparagine residues) attachments of simple or complex carbohydrates (e.g. monosaccharides, branched polysaccharides) to proteins. Upon *Hydroxylation*, hydroxyl groups are attached to proline residues. *Methylation* refers to transfer of methyl groups to arginine or lysine residues. *Nitrosylation* leads to the incorporation of nitric oxide into the thiol group of cysteine residues. Protein *phosphorylation* is associated with attaching phosphate groups to serine, threonine, or tyrosine protein residues. Both s*umoylation* and *ubiquitination* are characterized by the attachment of small proteins to target proteins modifying their function or stability or, in the case of ubiquitination, tagging them for degradation. While the previous 10 PTM-types are characterized by the attachment of chemical moieties to proteins, *disulfide bond* and *proteolytic cleavage* lead to posttranslational modifications via changing the chemical bond structure within a given protein and in the process removing atoms from it, either by forming a covalent bond (disulfide bond between two cysteine residues accompanied by the removal of two hydrogen atoms) or by breaking peptide bonds (proteolytic cleavage removing HOH). As the latter two met our established count-criteria and do indeed modify the protein, we retained them for the initial analyses. However, as data were available for human only, and to confine the analyses to moiety-addition-type PTMs, both PTM-types were left out in the subsequent analyses of network properties. For several PTM-types, several subtypes exist, e.g. Ser/Thr/Tyr phosphorylation. Generally, we considered PTM-types based on the added moiety, but also repeated selected analyzes with a further subsetting of the datasets based on the receiving group on the protein.

**Table 1:** Frequency table of proteins associated with the selected PTM-types and species.

**NCBI taxonomy ID**	**Species Name**	**acetylation**	**amidation**	**carboxylation**	**disulfide bond**	**glycosylation**	**hydroxylation**	**methylation**	**nitrosylation**	**phosphorylation**	**proteolytic cleavage**	**sumoylation**	**ubiquitination**
10090	*Mus musculus*	1688	7	11	0	1808	5	879	996	9770	0	67	5616
10116	*Rattus norvegicus*	303	25	14	0	164	8	198	85	3496	0	14	523
9913	*Bos taurus*	103	19	34	0	108	11	20	3	175	0	3	6
9606	*Homo sapiens*	4858	38	88	190	1823	33	1118	368	14425	388	327	6440
7227	*Drosophila melanogaster*	10	13	3	0	83	0	7	0	2415	0	0	2
6239	*Caenorhabditis elegans*	16	13	1	0	214	0	2	0	2138	0	0	0
3702	*Arabidopsis thaliana*	96	1	6	0	71	6	23	41	9198	0	5	51
4932	*Saccharomyces cerevisiae*	494	1	0	0	55	0	27	1	3195	0	17	125
4896	*Schizosaccharomyces pombe*	2	0	0	0	11	0	8	0	1072	0	0	6

Listed are the numbers of proteins with the corresponding PTM-type in the respective species.

The collected sets of proteins associated with the twelve different posttranslational modifications (PTMs) across the nine selected species were first investigated for PTM-type specific biological process involvement based on GO-term enrichment statistics as well as probed for significant co-occurrence patterns of different PTM-types on the same protein. Subsequently, we investigated whether protein sets associated with particular types of PTMs exhibit characteristic protein interaction network (PIN) properties. For the latter, we computed three basic and commonly used network properties, the degree, the clustering coefficient, and the closeness centrality, associated with proteins belonging to different PTM-specific protein sets when mapped onto the species-specific PIN. Finally, we tested whether pairs of interacting proteins exhibit preferences with regard to PTM-types they carry.

### PTM-specific biological process and location enrichment analysis

Frequently, proteins are modified not only by a single PTM event, but by several and of different PTM-types. Thus, if we wish to understand the role of individual PTM-types in the context of protein-protein interactions, we first need to understand their co-occurrence on the same protein as well as their functional profile as it seems plausible that PTM-types associated with similar functional involvement will also exhibit similar characteristics with regard to their protein interactions.

**Figure 1:**
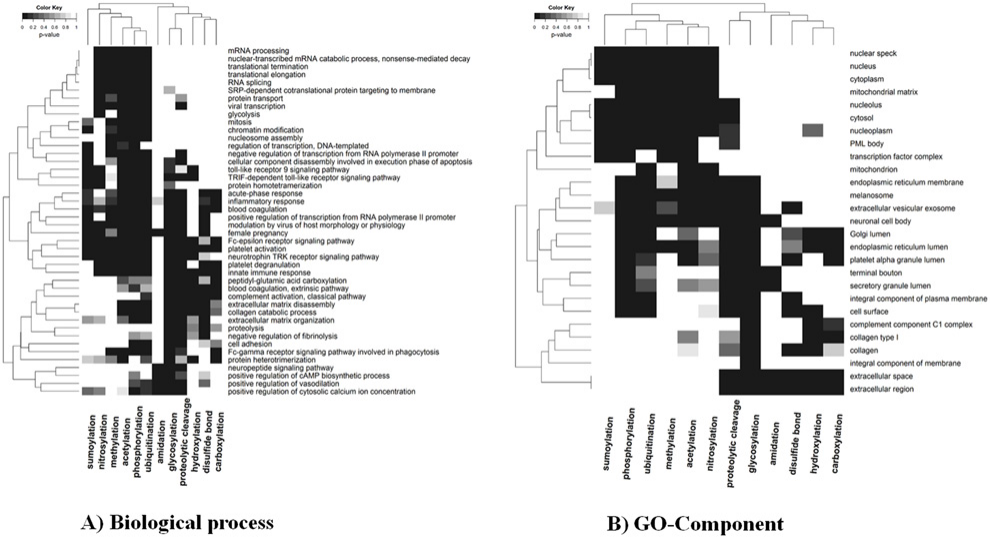
Heatmap of significant A) biological process and B) GO-Component terms across all studied PTM-types. The top five GO-terms were included that were found significantly enriched for each PTM-type. Each element in the heat map (Euclidean distance hierarchical clustering, average linkage) represents the grey-scale-encoded *p*-value, in which a particular combination of PTM-type and GO-term was found significantly enriched. The combined whole UniProtKB-GOA for all the selected species was used as the background set, Fisher’s exact test with FDR correction was used for the enrichment analysis, and the *p*-value (FDR) threshold indicating significance was set to 0.01.

The functional significance of specific PTM-types and the respective proteins carrying them has been amply investigated [26, 32, 36, 37]. To provide a comparative overview of the selected PTM-types studied here, we integrated all species-specific gene ontology (GO) annotations into a merged set and determined preferred biological process involvements, functional roles, and subcellular locations based on GO-enrichment statistics computed for this artificial “super species”. Consistently across all GO-term domains (process, function, and location), the 12 PTM-types are grouped into two major groups with sumoylation, nitrosylation, methylation, acetylation, phosphorylation, ubiquitination in one group (group-I), and disulfide bond, carboxylation, hydroxylation, proteolytic cleavage, glycosylation and amidation in the other (group-II) (Fig. 1, S1 Fig.). While group-I PTM-types were found preferentially in proteins located in the cytosol and nucleus and involved in regulatory processes (most noteworthy, transcriptional regulation), group-II PTM-types appear associated with membrane-, subcellular compartment localizations (carboxylation), extracellular locations and secretory processes. In line with several reported observations on their concerted action [38], phosphorylation and ubiquitination were found with similar GO process and location profiles. Acetylation appears to be involved in similar processes as well. Indeed, the combined action of these three PTM-types has been described in selected cases as, for example, for the protein p53 [20]. Furthermore, glycosylation and proteolytic cleavage exhibit similar GO-term characteristics, which may reflect the involvement of and even interplay between both PTMs in the modification of secreted and/or membrane-embedded proteins [39]. Thus, different PTM-types have similar functional involvement and location profiles suggesting that their characteristic protein-interaction network properties may also be similar, which appears implied in particular based on common localizations influencing the scope of potential protein interaction partners.

**Figure 2:**
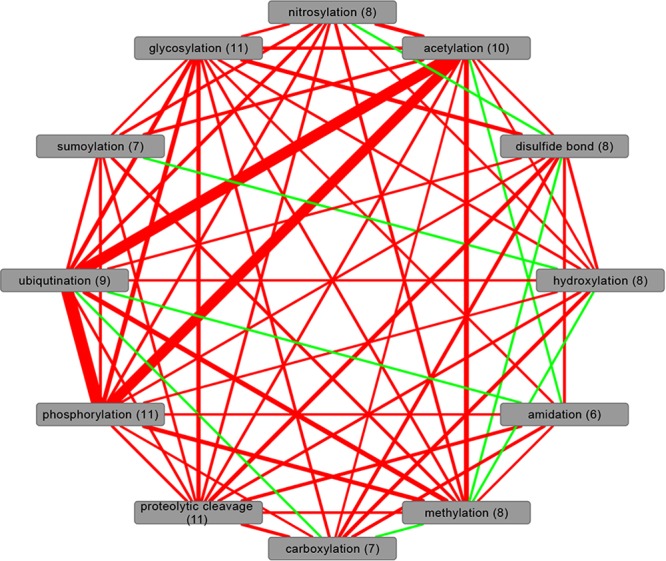
Co-existence network of PTM-types in the proteome of *Homo sapiens*. Nodes represent the protein sets associated with the different PTM-types. Edge width was set proportionally to the Jaccard index indicating the overlap between the different protein sets. Edge colors indicate significance with red highlighting PTM-pairs whose overlap was found significant based on Fisher’s exact test with FDR-adjusted *p*-value threshold set to 0.01, and green otherwise. Numbers in parentheses are the counts of significant “red” co-existence edges to other PTM-types.

**Figure 3:**
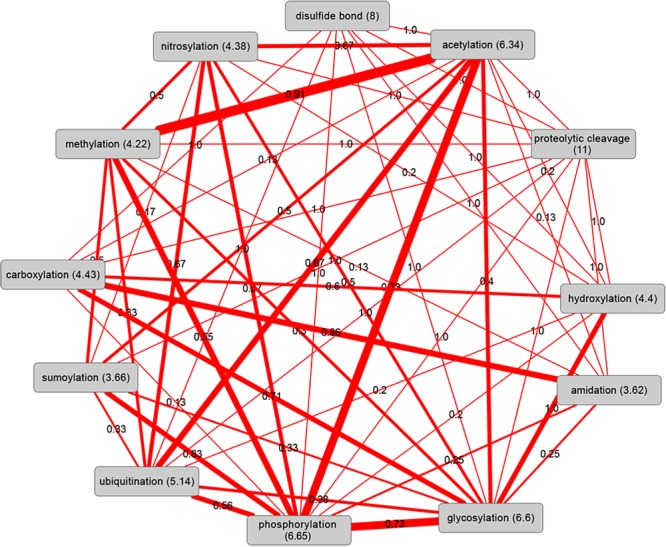
Significant co-existence pairs of PTM-types across all selected species. Edge width was set proportionally to the number of species in which a particular PTM pair was found to occur more frequently than expected (see legend to Fig. 2) at significance levels of FDR-corrected *p*-values<0.01. The values on the edges indicate the number of species with significant co-existence normalized by the number of common species between each pair of PTM-types as not all PTM-types are present in all species based on our filtering criteria (see Methods). Numbers in parentheses are the normalized counts of significant “red” co-existence edges to other PTM-types.

The co-occurrence patterns of different PTM-types on the same protein are critical confounding factors for the analysis of the individual PTM-types in the context of protein interactions. Evidently, frequent PTM-types will have a greater chance of co-occurring with other PTM-types on the same protein. Indeed, as judged by the Jaccard-distance, protein sets in human associated with phosphorylation, acetylation, and ubiquitination—the three PTM-types with the most observed instances (Table 1)—exhibit large overlaps (Fig. 2). However, as we tested for deviations from the expected chance overlap as well, this co-occurrence on the same protein also seems significant. In general, the overlap amongst all PTM-types studied here is extensive. The reduced overlap for amidation, disulfide bond, hydroxylation carboxylation, and methylation with other PTM-types appears largely caused by their low frequency. Similarly, when expanding the overlap analysis to all species considered here, a large overlap between phosphorylation and acetylation is evident (Fig. 3). Interestingly, when viewed across several species, methylation emerges as a PTM-type with significant co-occurrence with both acetylation and phosphorylation possibly reflecting their joint association with histones [40]. Furthermore, glycosylation and phosphorylation appear to frequently co-exist on the same protein.

In conclusion, the overlap of different PTM-types on the same proteins is extensive and greater than expected by chance. Even though suggested by the separate clustering of PTM-types based on their functional and location annotations (Fig. 1), with regard to their co-occurrence pattern, no equivalent segregation is apparent. Therefore, all PTM-types will—when analyzed jointly—likely exhibit similar protein-interaction characteristics. While primarily reporting results on the global protein sets (including overlaps), we also performed analyses on the one-PTM-type-only protein sets. Evidently, one cannot be certain that those unique sets are truly unique in reality as not all PTM-types and their instances have been identified yet. Furthermore, rendering the data set PTM-type specific, i.e. reducing the protein sets to sets conforming to one PTM-type only must inevitably lead to a massive reduction of statistical power.

### PTM-type specific protein interaction network properties

We inspected the protein sets associated with specific PTM-types in the context of known protein-protein interactions. We mapped all proteins with annotated PTMs onto the respective protein interaction networks (PINs) of the nine selected species (Table 2). By computing three network properties, the degree, the clustering coefficient and the closeness centrality, we wished to investigate whether proteins associated with particular PTM-types exhibit distinct interaction characteristics that may be indicative of a PTM-specific function. The *degree* quantifies the average number of connections a protein engages in. Thus, it reflects on how many interaction partners may be affected by a PTM of a given protein. The *clustering coefficient* allows estimating whether the proteins connected to a central reference protein are in turn connected amongst themselves. High clustering coefficients would indicate a closely knit network of local interactions, whereas low clustering coefficients would suggest that separate molecular processes with little communication between them are modulated, when a central protein undergoes a PTM. Finally, the *closeness centrality* allows assessing how centrally a particular protein resides relative to the overall network. Proteins with high closeness centrality are situated in central network positions such that they may serve central information relay functions. By contrast, low closeness centrality corresponds to peripheral locations as typical of initial receptor molecules. (For a formal definition of the three network properties, please see Methods.) Therefore, all three chosen network properties allow a direct interpretation of the specific function of PTMs with regard to impact (degree) and role as a potential information relay hub (clustering coefficient and closeness centrality) in the network of all interacting proteins in the cell.

**Table 2:** PIN size (number of proteins and interactions) for the nine selected species after excluding the components with the size less than 100.

**NCBI taxonomy ID**	**Species Name**	**STRING**		**IntAct**		**Common**	
		**Proteins**	**Interactions**	**Proteins**	**Interactions**	**Proteins**	**Interactions**
10090	*Mus musculus*	6599	55812	5983	14641	1068	1101
10116	*Rattus norvegicus*	3544	19394	1307	1866	180	156
9913	*Bos taurus*	2899	16119	113	183	68	80
9606	*Homo sapiens*	8949	71153	10588	57922	3592	7556
7227	*Drosophila melanogaster*	4319	50039	7280	24368	1052	1164
6239	*Caenorhabditis elegans*	3796	32247	3604	7785	684	719
3702	*Arabidopsis thaliana*	5528	34129	3499	8534	1186	2181
4932	*Saccharomyces cerevisiae*	4743	63169	5492	68891	3103	11723
4896	*Schizosaccharomyces pombe*	2748	22235	0	0	0	0

High confidence PINs extracted from STRING are listed in the “STRING” column, the “IntAct” column contains the PIN sizes extracted from IntAct. The “Common” column indicates the number of common proteins and interactions in STRING and IntAct. Only those proteins with available UniProt ID were considered.

**Figure 4:**
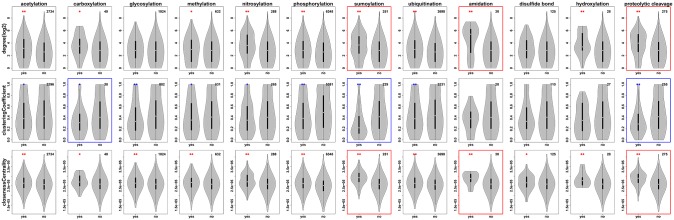
Protein interaction network properties of proteins associated with different PTM-types in *Homo sapiens*. The network property values of proteins annotated to undergo a particular PTM-type or not are shown by violin plots. The number at the top right corner of each graph represents the number of proteins with the corresponding PTM-type and valid network property definitions in *Homo sapiens*. Protein interactions were taken from the STRING database. The total numbers of proteins and associated number of interactions in *Homo sapiens* with confidence score>=0.9 were 8,949 and 71,153, respectively. The red (blue) asterisks at the top of violin plot represents the corresponding PTM group has a significantly higher (lower) median value compared to the non-PTM group (*: *p*-value 0.05, **: *p*-value 0.01) by Mann-Whitney test with FDR correction. The top 3 PTMs which have high percentage of median difference between PTM group and non-PTM group for each network property are highlighted with red (increased) or blue (decreased) margin.

Fig. 4 shows the frequency distributions of the PTM-type-specific network properties exemplified in *Homo sapiens*. Overall, all PTM-types exhibit a tendency to have higher degrees, lower clustering coefficients, and higher closeness centralities than protein sets not carrying the respective PTM-type, which includes a set of human proteins (1,864 or 20.8% of all human proteins) currently not known to undergo any of the 12 PTM-types considered here. The latter set (no PTM), was observed with lower degree, higher clustering coefficient, and lower closeness centrality than proteins undergoing a PTM (S2 Fig.). With regard to degree, the largest increases relative to the respective reference sets were observed for sumoylation, proteolytic cleavage, and amidation, albeit for the latter the count of observed instances is low. By contrast, glycosylation shows almost no change of degree relative to its reference set. With regard to the clustering coefficient, sumoylation, proteolytic cleavage, and carboxylation were found with the largest decreases relative to their respective control sets. Finally, sumoylation, proteolytic cleavage, and amidation were the top-three PTM-types associated with the largest relative increase in their median closeness centrality compared to their proteins sets devoid of the respective PTM-type. Again, glycosylation was found with the smallest relative change with regard to closeness centrality. Thus, excluding the PTM-types with very low counts (amidation and carboxylation), sumoylation and proteolytic change were identified as the two PTM-types associated with the largest relative differences across all three network properties examined. In short, both are characterized by high degree, low clustering coefficient, and high closeness centrality. Glycosylation is found at the other end of the spectrum with no change with regard to degree and closeness centrality, but a drop in clustering coefficient. The three most abundant PTM-types in human—based on available data—acetylation, phosphorylation, and ubiquitination, all show significant and comparable degree and closeness centrality increases. With regard to closeness centrality, phosphorylation is signified by the largest drop among the three PTM-types relative to its control protein set, while the other two show a smaller (ubiquitination) or no change (acetylation).

**Figure 5:**
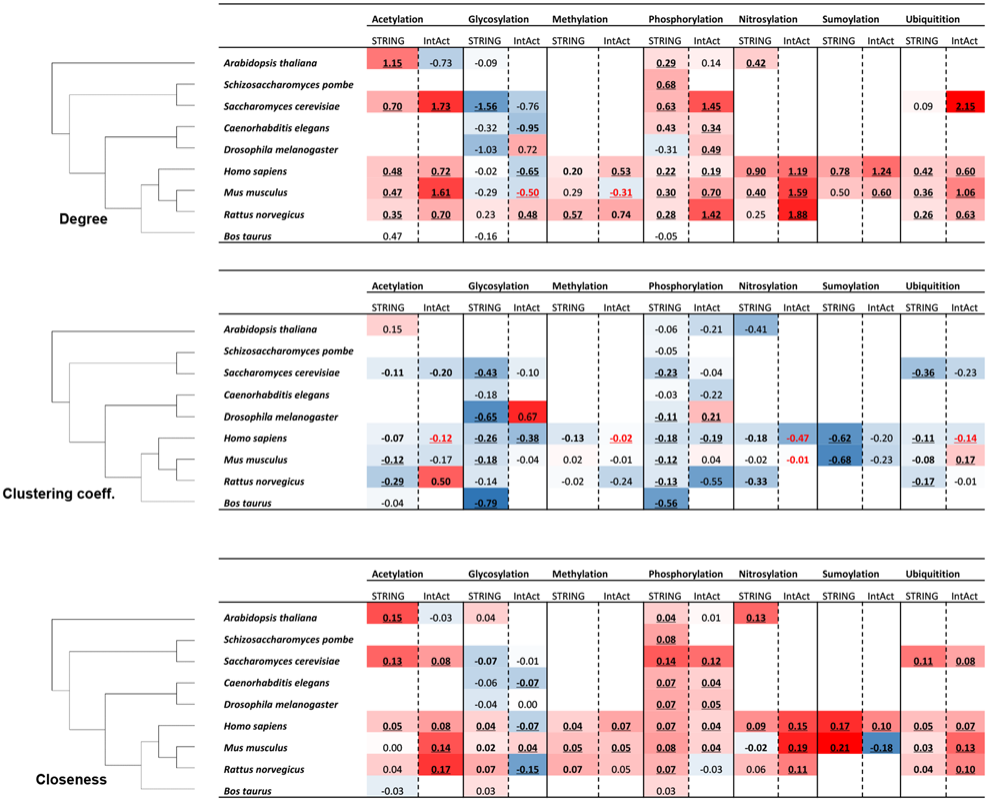
Degree, clustering coefficient, closeness centrality analysis for proteins with different PTM-types in each considered species and associated high-confidence STRING and IntAct PIN. The species are ordered according to their phylogenetic relationships as shown on the left. For every PTM-type, the log-2 of fold difference value for the degree/clustering coefficient/closeness centrality value relative to the respective value associated with proteins not carrying this particular PTM-type are given for PINs based on STRING and IntAct, respectively. Color scale indicates increased (red) or decreased (blue) values in the PTM-set relative to the non-PTM-set with symmetric color intervals (i.e. full color saturation based on the maximal absolute increase or decrease fold difference observed across all values in the table.) Bold-font (underlined) fold-changes indicate significant fold-changes at *p*<0.05 (*p*<0.01) by Mann-Whitney test with FDR correction, the values in red or blue text represent significantly higher or lower network properties, which are inconsistent with the background color based on mean (not median) values. PTM-types “carboxylation”, “proteolytic cleavage”, “hydroxylation”, and “disulfide bond” are not included in this analysis as associated numbers were available for *Homo sapiens* only.

Next, we expanded our analyses to the remaining eight species considered here (Fig. 5). To avoid database specific effects, we considered two sources of PIN information, STRING and IntAct [41, 42]. While STRING contains integrated interactions from different sources, IntAct contains experimentally verified interactions extracted from literature and based on direct user submissions. Except for a few cases (13 out of 86 with data available for both PIN-resources), consistent results across the two PIN-data resources were obtained. Furthermore, for some of the IntAct derived PIN-properties, we detected a difference in their mean value compared to their median, reflecting the lower counts of IntAct events resulting in non-Gaussian/asymmetric distribution. Furthermore, truly comparing the different PTM-types across several species is possible only for the PTM-types acetylation, glycosylation, and phosphorylation, as for the others sufficient PIN-information is lacking. Also, we restricted the analyses to the addition-type PTM-types with sufficient data acetylation, glycosylation, methylation, phosphorylation, nitrosylation, sumoylation, and ubiquitination.

Despite these data limitations, a similar overall picture emerges as observed in human. All examined PTM-types appear to be associated with proteins with increased degree and closeness centrality, but decreased clustering coefficient relative to protein sets not carrying the respective PTM-type. Glycosylation is a notable exception and seems associated with slightly decreased, rather than increased, degree centrality, while exhibiting similarly decreased clustering coefficients across all species as the other PTM-types. With regard to closeness centrality, the results for glycosylation are mixed with a few species (the four mammalian species and the plant *Arabidopsis thaliana*) showing slightly increased values for STRING-based PINs and decreased values in the remaining species. However, clearly more table cells are colored blue signifying lowered values than for the other PTM types further supported by negative fold-change values for IntAct PINs. Thus, overall, glycosylation appears to generally be associated with no significantly changed, or slightly lowered closeness centrality relative to control protein sets.

Several of the PTM-types inspected here can be subdivided further into separate sets based on the identity of the targeted group on the protein (Lysine vs. N-terminal acetylation, N- or O-linked glycosylation, arginine-/lysine-methylation, and serine/threonine and tyrosine phosphorylation. Especially in the case of S/T vs. Y-phosphorylation differences in the associated PIN-properties would be of interested given the importance of phosphorylation in general, and in particular, as the two different types are catalyzed by different kinases [43]. However, when subdividing the protein sets into the target-specific PTM types, consistent results were obtained as reported for the merged sets (S3 Fig.). As the only notable exception, the difference in degree observed for O-linked glycosylation (tendency for increased degree relative to reference set) compared to N-linked glycosylation (trending towards decreased degree) is worth mentioning.

In an attempt to address a possible confounding influence of co-occurring different PTM-types on the same protein, we repeated the analysis of PTM-type-specific PIN properties shown in Fig. 5 for sets of proteins that are annotated to undergo one PTM-type only (S4 Fig.). Inevitably, this dramatically reduced the number of proteins that can be used (S1 Table). Hence, a meaningful analysis was possible for four PTM-types (acetylation, glycosylation, phosphorylation, and ubiquitination) only. Again, some conflicts between STRING and IntAct derived results render drawing clear conclusions difficult. However, phosphorylation again comes out as being associated with high-degree, low clustering coefficient, and high closeness centrality proteins compared to reference unphosphorylated protein sets. Glycosylation, on the other hand, is found again with low degree, low clustering coefficient, and low closeness centrality compared to control sets of proteins that are not glycosylated. Acetylation and ubiquitination both appear less consistent with the results reported for the whole protein set (Fig. 5). Ubiquitination was found with low degree, high clustering coefficient, and low closeness centrality; i.e. opposite the trend reported in Fig. 5. For acetylation, no clear trends are evident also because of many conflicts between STRING and IntAct based results. Thus, PIN-properties for phosphorylation and glycosylation are confirmed in the unique protein sets, whereas acetylation and ubiquitination either behave differently when protein sets are properly reduced to unique sets, or clear conclusions cannot be drawn as of yet because of data limitations.

### Cross-protein PTM interaction patterns

**Figure 6:**
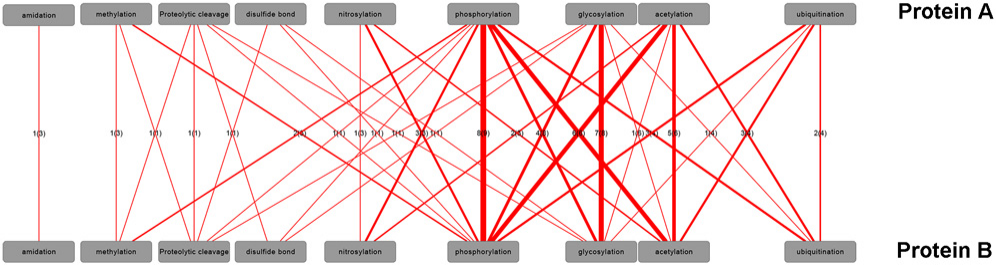
Pairwise interactions of proteins carrying different PTM-types (one-PTM-type-only dataset, see Methods). Number of species with statistically increased frequency of protein-protein interactions (designated as protein A and B, respectively) carrying the respective PTM-types. Linewidth is set proportional to the number of species (indicated as edge labels), which exhibit significant interactions of PTM-types carried by interacting proteins. The value in the parentheses corresponds to the number of common species with available PTM and PIN information. The contingency table for the Fisher exact test contained the respective counts for number of proteins associated with a particular PTM-pair versus all alternative pairings and whether they have been reported to interact or not with FDR-corrected p-value <0.01. Note that the counts of pairwise interactions between protein A and B are by definition symmetric. Hence, labels were added to one direction only. Sumoylation, hydroxylation, and carboxylation were left out because no related significant interactions were found.

Above, we examined the co-occurrence of different PTM-types detected on the same protein (Fig. 3). We extended the co-occurrence analysis to pairwise physically interacting proteins carrying different PTMs based on protein sets characterized by one PTM-type only (Fig. 6). For all PTM-types but disulfide-bond proteins, there is a tendency to self-interact, i.e. two separate proteins carrying the same PTM-type interact more often than randomly expected. Phosphorylated proteins display the broadest interaction range with significantly more interactions than expected to 8 other PTM-type proteins, followed by glycosylated proteins (6 distinct PTM-type partners), and acetylation (5 distinct PTM-type partners). By contrast, proteins associated with methylation, disulfide-bond formation or amidation exhibit a reduced interaction spectrum with likely interactions to only three of fewer other PTM-type proteins including interactions between two proteins carrying the same PTM-type. Acetylation, glycosylation, and phosphorylation form a clique of more than expected interactions among these three PTM-types. Especially phosphorylated proteins were found to interact more often with acetylated proteins than expected by chance. Please note that the analysis displayed in Fig. 6 is controlled for abundance; i.e. the reported interactions are above the expected chance-encounters. Interaction statistics including proteins including those with multiple different PTM-types is provided as S5 Fig. As frequently, proteins carry multiple different PTM-types, conclusions with regard to preferred cross-protein PTM-type interactions is less meaningful. However, the trends described above are apparent as well.

### Disease association of PTM-type-specific network properties in human

We tested whether proteins are more likely to be implicated in human disease when their associated PIN property values were at the high or low end of the spectrum. Most significantly for phosphorylation, but also evident for ubiquitination, acetylation, and glycosylation, we detected a larger than expected overlaps with known human disease proteins for high-degree proteins only, but not for proteins undergoing the same PTM-type but with low interaction degree proteins. Similarly, glycosylated and phosphorylated proteins are more likely disease associated when they have high closeness centrality. By contrast, low clustering coefficient appears correlated with disease association for proteins undergoing phosphorylation, acetylation, and ubiquitination (Table 3).

**Table 3:** Association of PTM-type and PIN-property specific protein sets with known human disease proteins.

**PTM-type**	**degree**	**clustering coefficient**	**closeness centrality**
acetylation	**4.94E-07**	***2.52E-03***	9.94E-01
amidation	1.00E+00	1.00E+00	1.00E+00
carboxylation	1.57E-01	9.36E-01	6.37E-02
disulfide bond	9.49E-02	1.00E+00	5.05E-01
glycosylation	**1.04E-05**	2.42E-01	**8.15E-05**
hydroxylation	1.00E+00	3.46E-01	1.00E+00
methylation	**4.35E-02**	1.00E+00	1.00E+00
nitrosylation	1.00E+00	1.00E+00	7.10E-01
phosphorylation	**9.41E-17**	***4.94E-07***	**2.89E-03**
proteolytic cleavage	**4.71E-02**	7.16E-01	6.62E-02
sumoylation	2.42E-01	5.05E-01	**4.71E-02**
ubiquitination	**5.06E-09**	***3.70E-03***	2.10E-01

Protein sets were sorted in descending order of the respective PIN-property and the top/bottom 25% tested for overlap with human disease genes reported in OMIM (http://www.ncbi.nlm.nih.gov/omim) based on a Fisher’s exact test with FDR-correction with respective counts of proteins binned according to binary decisions top/bottom 25% property and disease association yes/no forming the 2×2 contingency table. *p*-values highlighted red indicate significant overlap of the top-25% set of proteins for a particular network property with human disease proteins, while blue values signify larger than expected overlaps of the bottom-25% protein sets with proteins annotated to be involved in disease processes at p-value thresholds of *p*-value 0.05, 0.01 (if underlined), respectively. Black font color indicates no significant overlap of neither the top nor the bottom 25% set.

## Discussion

The modulation of protein function via different types of post-translational modifications (PTMs) and their combinatorial interplay has attracted considerable attention in recent years [15–27]. In this study, we added the interaction layer to the study of PTMs by performing a systematic investigation of the network properties of the different PTM-types in the context of the physical interactions of PTM-carrying proteins. For twelve different PTM-types and across nine diverse species, we determined characteristic and informative network parameters with the goal to investigate whether particular PTM-types are associated with specific and possibly “strategic” placements in the context of all protein interactions such that their individual role in the orchestration of the combined action of all proteins becomes apparent.

Generalized across all PTM-types and species investigated here, PTM-carrying proteins appear engage in more physical contacts, with a reduced clustering coefficient among those proteins they are interacting with, and elevated closeness centrality than their respective protein sets devoid of the particular PTM-type (Fig. 5) or that, as far as we currently know, do not harbor any PTM of any type (S2 Fig.). Differences between the twelve studied PTM-types proved less pronounced with essentially all—except for glycosylation (see below)—following the same trend of high degree, low clustering coefficient, and high closeness centrality with only subtle differences in magnitude between them. However, given the present data coverage, it is not yet possible to conclusively decide whether these differences are statistically significant and biologically relevant. When further subsetted into special types of PTMs (e.g. S/T/Y phosphorylation), no significant sub-type differences were evident (S3 Fig.). As motivated above, the three selected network properties were selected specifically to allow conclusions as for the “strategic” roles of PTM in the context of interactions. According to this logic, proteins with PTMs engage in more and different process than non-PTM proteins and play central information relay functions.

Focusing on human PIN and PTM data, sumoylation and proteolytic cleavage stand out as being associated with the largest relative increase of degree and closeness centrality relative to reference sets. Proteolytic cleavage has been associated with activation processes and protein targeting events (cleavage of targeting N-terminal peptide) and constitute a “dramatic” modification as the relative change of molecular composition of a protein can be significant. Furthermore, transporting proteins to different compartments will inevitably influence the possible interaction scope. The significance of sumoylation in a range of regulatory processes has been increasingly recognized [44]. Our results underscore the importance of this PTM-type.

Phosphorylation, the PTM-type with the largest data support, was identified as the PTM-type with the consistently central and with the largest potential influence scope (Fig. 5). Phosphorylated proteins reside in central network positions (high closeness centrality) and interact with many other proteins (high degree) including specifically pairwise interactions with proteins carrying any of the other four PTM-types as well as other phosphorylated proteins (Fig. 6 — pairwise interaction figure). Examples from human of phospho-proteins interacting with proteins carrying other PTM-types include the kinases: mitogen-activated protein kinase 1 (MAPK1), interleukin-1 receptor-associated kinase 2 (IRAK2), and spleen tyrosine kinase (SYK). Those proteins each interact with other proteins representing four different PTM-types. These findings underscore once again the central importance of phosphorylation as perhaps the most important and central PTM-type identified so far. Similar characteristics were found for acetylation, albeit the detected magnitude and statistical support is lower.

By contrast, glycosylation was found associated with proteins of low degree, low clustering coefficient, and low closeness centrality (Fig. 5). In particular the low degree and low closeness centrality of glycosylated proteins may be interpreted as consistent with their preferred location in cytosolic membranes and to act as receptors and cell-cell communication mediators (Fig. 1, GO-term clustering) [45]. Unlike the other four PTM-types, the transferred glycosyl-groups can be large leading to impeded protein-protein interactions of glycosylated proteins. In addition, because of their frequent embedding in membranes, they operate in two dimensions, not three as for soluble cytosolic proteins, effectively cutting down the interaction potential.

As shown in Fig. 6, all PTM-types are found on proteins that exhibit a tendency to interact with other proteins carrying the same PTM-type. In the case of phosphorylation, such interactions are interpretable as the well known as phosphorylation/kinase cascades [46, 47]. It is also possible that the detected tendency of PTM-types to self-interact originates from protein complexes, in which all partners undergo the PTMs of the same type. For example, in histone complexes, lysine residues on different proteins are acetylated modifying the binding affinity of histones to DNA [48, 49]. Similar consideration apply to methylation events in histone [50] and other protein complexes [51].

By including nine species from different kingdoms and lineages, we aimed to extract both general and species/lineage-specific trends. However, currently available datasets proved comprehensive enough for a few species only (human, mouse, rat). In the case of phosphorylation, sufficient data were available across all nine species and provided a consistent result of increased degree and closeness centrality and a decreased clustering coefficient (Fig. 5).

The increased likelihood of a functional association of proteins with high interaction degree and their involvement in human disease has been reported before [52, 53]. In selected cases, proteins carrying PTMs have also been reported to be more likely related to disease processes than non-PTM proteins [54–56]. Our dataset allowed us to expand this analysis to testing specific PTM-types combined with their PIN-characteristics. Our results suggest that not only does a PTM render proteins more likely disease associated, but that this association may depend on what PIN context it is embedded in. High degree, low clustering coefficient, and high closeness centrality proteins are more likely to be disease associated (Table 3) than their respective counterpart sets at the respective other end of the property PIN-property spectrum, especially for the PTM-types phosphorylation and glycosylation, albeit it for the latter, no significant clustering coefficient trend was detected. Examples of disease-associated phosphorylated or glycosylated proteins detected with high degree and closeness centrality or low clustering coefficient are provided in Table 4. It may be speculated that proteins with the properties identified as more likely disease associated based on their PIN properties may constitute promising candidates for intensified research. Evidently, the relevance of the protein p53 in human cancer development has long been recognized [57]. In our study, it was identified as one with characteristic network properties typical of disease associated proteins in general.

**Table 4:** Examples of human PTM-carrying proteins with characteristic PIN-properties and their known disease involvement.

**PTM-type**	**PIN- characteristics**	**Protein**	**Ensembl ID**	**Disease**
Glycosylation	high degree, high closeness centrality	Pro-epidermal growth factor	ENSP00000265171	Hypomagnesemia 4 (HOMG4) [MIM:611718][67]
		Transforming growth factor beta-1	ENSP00000221930	Camurati-Engelmann disease (CE) [MIM:131300][68]
		Interleukin-6	ENSP00000258743	Rheumatoid arthritis systemic juvenile (RASJ) [MIM:604302][69]
Phosphorylation	high degree, high closeness centrality	Cellular tumor antigen p53	ENSP00000269305	Esophageal cancer (ESCR) [MIM:133239] [70]; Li-Fraumeni syndrome (LFS) [MIM:151623][71]
		RAC-alpha serine/threonine-protein kinase	ENSP00000270202	Breast cancer (BC) [MIM:114480]; Colorectal cancer (CRC) [MIM:114500] [72]; Proteus syndrome (PROTEUSS) [MIM:176920] [73]
		Histone acetyltransferase p300	ENSP00000263253	Rubinstein-Taybi syndrome 2 (RSTS2) [MIM:613684][74]
Phosphorylation	low clustering coefficient	Autoimmune regulator	ENSP00000291582	Autoimmune polyendocrine syndrome 1, with or without reversible metaphyseal dysplasia (APS1) [MIM:240300] [75]
		ALK tyrosine kinase receptor	ENSP00000373700	Neuroblastoma 3 (NBLST3) [MIM:613014] [76]
		Ataxin-2	ENSP00000366843	Spinocerebellar ataxia 2 (SCA2) [MIM:183090] [77]; Amyotrophic lateral sclerosis 13 (ALS13) [MIM:183090] [78]

Evidently, this study hinges on the completeness and accuracy of the available PTM and PIN data as well. Any bias towards a specific detection of particular protein classes and their associated PTM may further skew our results. By imposing a high significance cutoff for the PIN-data (confidence score > 0.9), and furthermore exploiting two data sources (STRING and IntAct), we believe to have taken proper precautionary steps even though some discrepancies were detected (Fig. 5). However, at this point it cannot be decided whether the size of the dataset (relatively small IntAct data set) or the type of PINs that are recorded cause these differences. With regard to PTMs, we used experimentally verified PTMs only. Future investigations of the PIN characteristics of PTMs will benefit from the expected significant increase of experimentally verified sites. In addition, a larger set of different PTMs with sufficient numbers will likely become available, allowing also to further specify the PTM-types used in this study.

A possible selection bias may also come from preferentially profiling those proteins for PTMs that possess “interesting” properties such has high degree. However, as PTMs are increasingly identified in massive, “shotgun” style omics studies, such selection bias may not be that critical. Rather, abundance may be a concern then. However, for phosphorylation it was reported that protein abundance is not correlated with network properties [34, 35]. Furthermore, we also found that network properties are largely independent of the number of PTMs on a given protein (S6 Fig.). While significant due to the large number of observations, no relevant correlation was found neither for degree and nor clustering coefficient with the number of phosphorylation sites taken as the PTM-type with the largest available dataset. However, for closeness centrality, a more sizable positive correlation (*r* = 0.164) was detected suggesting that more heavily phosphorylated proteins occupy more central positions in the network of protein-protein interactions.

In conclusion, proteins carrying different types of PTMs differ from average non-PTM-proteins and differ between each other with regard to their protein interaction characteristics. Thus, their location within the web of physical protein-protein interactions is not only non-random, but very likely indicates their specific functional roles in the orchestration of molecular processes mediated by the physical interactions between proteins.

## Materials and Methods

### Datasets

**Post-translational modifications.** Post-translational modifications annotated as “experimentally verified” and the associated proteins were extracted from UniProt [58], PhosphoSitePlus [59], dbPTM [9], Phospho.ELM [12], PhosphoGRID [60], PHOSIDA [13], HPRD [61], OglycBase [62], PhosPhAt [14], P3DB [63], PTMcode [11] as of 2014 April. Subsequently, the sets obtained from the different data sources were consolidated to create a single set based on protein sequence position information. Initially, only those PTM-types were considered further, for which more than 1000 sites were reported across the various data resources, regardless of species. Subsequently, only those species were retained for which the count of PTM-sites across all PTM-types was 1000 or more. The intersection of both sets yielded the primary PTM-type-species dataset for analysis. To remove outliers, extremely long or short proteins as determined by falling below the 1-percentile or above the 99-percentile of observed protein sequence length distribution were removed. Furthermore, proteins with an extremely high number of PTM sites greater than or equal to. average(number of PTM sites)+3*sd(number of PTM sites)) were discarded as well. Twelve PTM types met those criteria in at least one species including 10 PTM-types in which a chemical moiety is attached: acetylation, amidation, carboxylation, glycosylation, hydroxylation, methylation, nitrosylation, phosphorylation, sumoylation, ubiquitination, and two PTM-types that modify the protein via forming or breaking bonds within the protein: disulfide bond and proteolytic cleavage. Nine species met those criteria for at least one PTM-type including representatives from the animal (mammals: *Mus musculus, Rattus norvegicus, Bos taurus, Homo sapiens*; insects: *Drosophila melanogaster, invertebrates: Caenorhabditis elegans*), plant (*Arabidopsis thaliana*), and fungal (*Saccharomyces cerevisiae, Schizosaccharomyces pombe)* kingdom, respectively (Table 1).

Many proteins carry more than one PTM-type. Some analyses were meaningful only if the considered proteins carry one PTM-type only (e.g. interaction between proteins carrying different PTM-types). Then, protein sets were filtered further and referred to as “one-PTM-type-only” (S1 Table). For statistics of the associated sub-sets of the dataset, see S2 Table.

**Protein-protein interaction networks.** For the nine species selected based on available PTM information, high confidence (confidence score>=0.9) protein-protein interactions (PINs) were extracted from STRING (version 9.05) [41]. In order to avoid database biases, each species’ protein-protein interactions were also extracted from the IntAct database (downloaded on 9.26.2013) [42], which stores interactions derived from literature curation or direct user submissions. To remove isolated interactions significantly affecting some of the network properties (e.g. closeness centrality), the network components with the size less than 100 were excluded (S3 Table). Only one component (the “giant”) component was left for each selected species. The sizes of the different PINs (species and data source) are summarized in Table 2. As the overlap between the STRING and IntAct interaction set is relatively small, the two datasets can be seen as largely disjoint and independent.

**Network properties.** The PINs associated with the selected species correspond to undirected networks. The *degree* of a node *n* is the number of edges linked to *n* [64]. The *clustering coefficient* [64] of a node *n* is defined as. C_*n*_ = 2e_*n*_/(k_*n*_(k_*n*_ - 1)), where k_*n*_ is the number of neighbors of *n* and. e_*n*_ is the number of connected pairs between all neighbors of *n*. It reflects the connectivity of adjacent nodes. The *closeness centrality* C_*c*_(n) of a node *n* is defined as the reciprocal of the sum of shortest path length originating from *n* to all other nodes *m* [64]: $C_{c}\left(n\right)=\frac{1}{\Sigma_{m}L\left(n,m\right)}$, where L(n,m) is the length of the shortest path between two nodes *n* and *m*. It ranges between 0 and 1. It corresponds to the inverse of the number of steps needed to traverse from all other nodes in the network to a selected node. The R package “igraph” (http://cran.r-project.org/web/packages/igraph/index.html) was used to compute the above mentioned network properties.

### Protein interaction network characteristics tests

For each PTM-type and across the selected species, the three selected network properties (degree, clustering coefficient, and closeness centrality) were computed for all proteins carrying the respective PTM-type and compared to those proteins not carrying this particular PTM-type. Significant differences between the respective two distributions were detected based on a Mann–Whitney test. The *p*-values were corrected for multiple testing considering as the total number of tests all tests across species, all PTM-types for each network property. In all cases of multiple testing correction, the FDR method was used [65]. Significance testing was applied to only those PTM-types and species with 30 proteins instances or more.

### Biological function enrichment analysis

For all species selected in this study, the available genome gene ontology (GO) process, function and cellular compartment annotations were extracted from UniProtKB-GOA [66] as the reference set. All the selected species were combined into one ‘species’ in the enrichment analysis. The method “elim” and the Fisher’s exact test with FDR were used for enrichment analysis using the “topGO” R package. The cutoff *P*-value was set to 0.01.

### Jaccard index for PTMs co-existence

For each pair of PTMs (A and B) in one species, The Jaccard index for the co-existence of A and B is defined as |Intersection(S_**A**_,S_**B**_)|/|Union(S_**A**_,S_**B**_)|, where the set of proteins associated with A and B are denoted as S_**A**_ and S_**B**_. Fisher’s exact test was used to test the significance of co-existence.

### Fisher’s exact test for cross-protein PTM interaction

For each pair of PTM-types and across the selected species, Fisher’s exact test was designed to test the over and under protein interaction frequency. The *p*-values were corrected for multiple testing considering as the total number of tests across all pair of PTM-types for each species. The cutoff *p*-value was set to 0.01. Significance testing was applied to only those pairs of PTM-types in each species with at least 10 proteins instances or more separately.

### Overlap between human disease proteins and PTMs associated proteins

Human disease proteins were downloaded from OMIM (http://www.ncbi.nlm.nih.gov/omim) as of May, 2014. In order to test the overlap between human diseases proteins with proteins associated with high (top 25%) or low (bottom 25%) network property values, Fisher’s exact test was used for all PTM-types. The p-values were corrected for multiple testing (FDR) across all PTM-types and network properties.

## Supporting Information

S1 Fig Heatmap for significant molecular function terms across all studied PTM-types. The top five GO-terms were included that were found significantly enriched for each PTM-type. Each element in the heat map (Euclidean distance hierarchical clustering, average linkage) represents the grey-scale-encoded p-value, in which a particular combination of PTM-type and GO-term was found significantly enriched. To he combined whole UniProtKB-GOA for all the selected species was used as the background set, Fisher’s exact test with FDR correction was used for the enrichment analysis, and the *p*-value (FDR) threshold indicating significance was set to 0.01.
(TIF)

S2 Fig Protein interaction network properties associated with proteins without any and with a PTM in *Homo sapiens*. The red (blue) asterisks on the top of violin plot represents the corresponding non-PTM group has a significantly higher (lower) median value compared to the non-PTM group (*: *p*-value 0.05, **: *p*-value 0.01) according to a Mann-Whitney test.
(TIF)

S3 Fig Degree, clustering coefficient, closeness centrality analysis for proteins with different PTM-subtypes in each species and associated high-confidence STRING and IntAct PIN (refined dataset). The species are ordered according to their phylogenetic relationships as shown on the left. For every PTM-type, the log-2 of fold difference value for the degree/clustering coefficient/closeness centrality value relative to the respective value associated with proteins not carrying this particular PTM-type are given for PINs based on STRING and IntAct, respectively. Color scale indicates increased (red) or decreased (blue) values in the PTM-set relative to the non-PTM-set with symmetric color intervals (i.e. full color saturation based on the maximal absolute increase or decrease fold difference observed across all values in the table.) Bold-font (underlined) fold-changes indicate significant fold-changes at *p*<0.05 (*p*<0.01) by Mann-Whitney test with FDR correction, the values in red or blue text represent significantly higher or lower network properties which are inconsistent with the background color.
(TIF)

S4 Fig Degree, clustering coefficient, closeness centrality analysis for proteins with different PTM types in each species and associated high-confidence STRING and IntAct PIN. Protein sets were selected to contain one PTM-type only (one-PTM-type-only dataset). The species are ordered according to their phylogenetic relationships as shown on the left. For every PTM-type, the log-2 of fold difference value for the degree/clustering coefficient/closeness centrality value relative to the respective value associated with proteins not carrying this particular PTM-type are given for PINs based on STRING and IntAct, respectively. Color scale indicates increased (red) or decreased (blue) values in the PTM-set relative to the non-PTM-set with symmetric color intervals (i.e. full color saturation based on the maximal absolute increase or decrease fold difference observed across all values in the table.) Bold-font (underlined) fold-changes indicate significant fold-changes at *p*<0.05 (*p*<0.01) by Mann-Whitney test with FDR correction, the values in red or blue text represent significantly higher or lower network properties, which are inconsistent with the background color based on mean (not median) values.
(TIF)

S5 Fig Pairwise interactions of proteins carrying different PTM-types (full dataset). Increased frequencies of protein-protein interactions (designated as protein A and B, respectively) carrying the respective PTM-types relative to expectation. Line width is proportional to the number of species, which exhibit significant interactions of PTM-types carried by interacting proteins. The contingency table for the Fisher exact test contained the respective counts for number of proteins associated with a particular PTM-pair versus all alternative pairings and whether they have been reported to interact or not with applied FDR-corrected p-value threshold of <0.01.
(TIF)

S6 Fig Scatterplot between the number of PTMs, here phosphorylation sites, and corresponding network properties (degree, clustering coefficient, and closeness centrality) for phosphorylated proteins based on the STRING network (the giant component was considered only) of *Homo sapiens*. The red line connects the mean network property values of proteins associated with different numbers of PTMs. Associated Pearson linear correlation coefficients, *r*, (and p-values) were: degree *r* = 0.056 (1.46E-06), clustering coefficient: *r* = -0.068 (6.27E-08), closeness centrality: *r* = 0.164 (0.00).
(TIF)

S1 Table Frequency table of proteins associated with the selected PTM-types and species after excluding proteins with more than one PTM-type (one-PTM-type-only dataset); i.e. every protein was annotated to harbor one PTM-type only. (DOCX)

S2 Table Frequency table of proteins associated with the selected PTM-subtypes and species (PTM-subtypes dataset). (DOCX)

S3 Table PIN size (number of proteins and interactions) for the nine selected species before excluding the components of size less than 100. (DOCX)
