# Supplementary figures and images for: The Roles of Post-translational Modifications in the Context of Protein Interaction Networks
Source: PLoS Comput Biol. 2015 Feb 18;11(2):e1004049. doi: 10.1371/journal.pcbi.1004049 (PMC4333291; doi:10.1371/journal.pcbi.1004049)

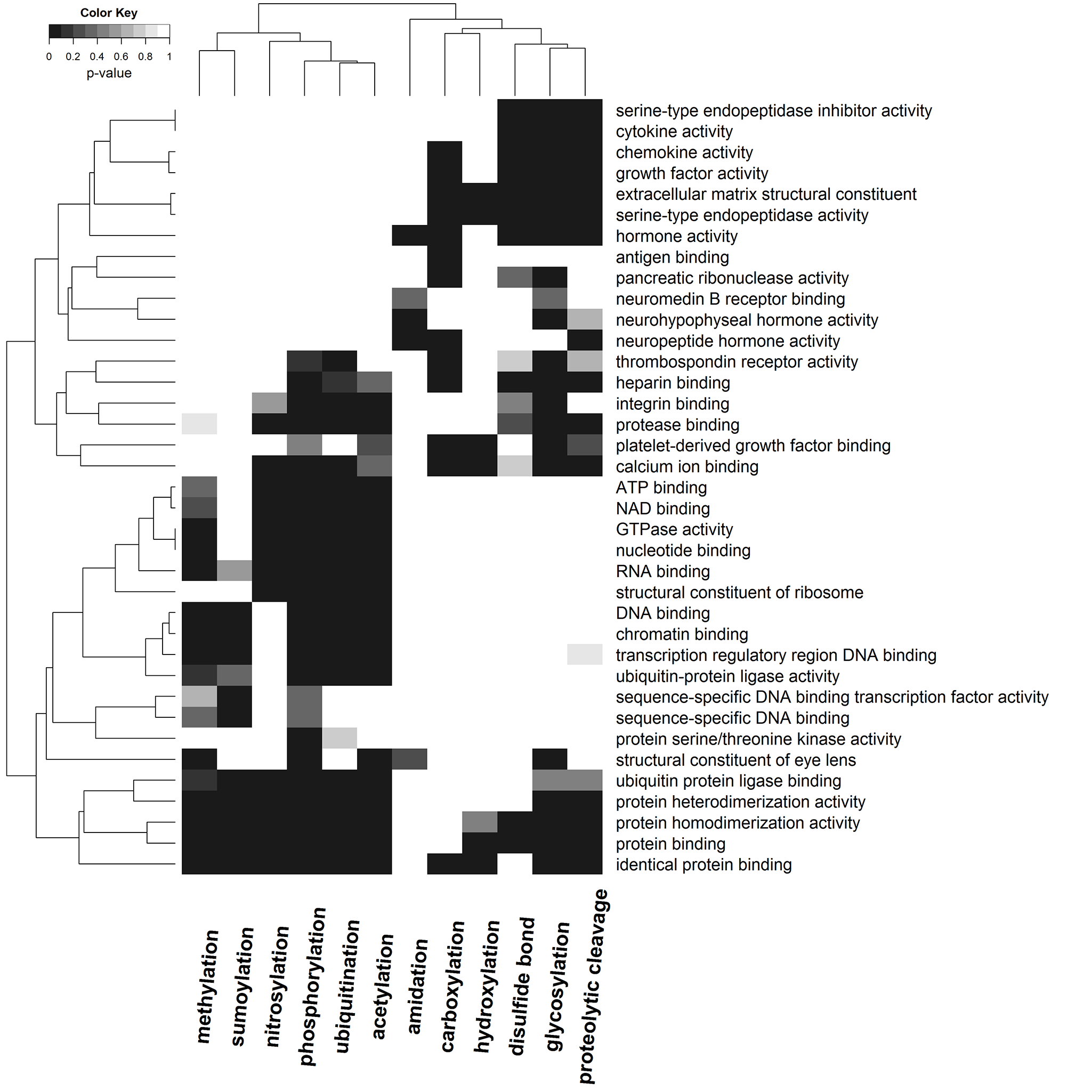

Supplement: S1 Fig — The top five GO-terms were included that were found significantly enriched for each PTM-type. Each element in the heat map (Euclidean distance hierarchical clustering, average linkage) represents the grey-scale-encoded p-value, in which a particular combination of PTM-type and GO-term was found significantly enriched. To he combined whole UniProtKB-GOA for all the selected species was used as the background set, Fisher’s exact test with FDR correction was used for the enrichment analysis, and the p-value (FDR) threshold indicating significance was set to 0.01. (TIF) [file pcbi.1004049.s001.tif]

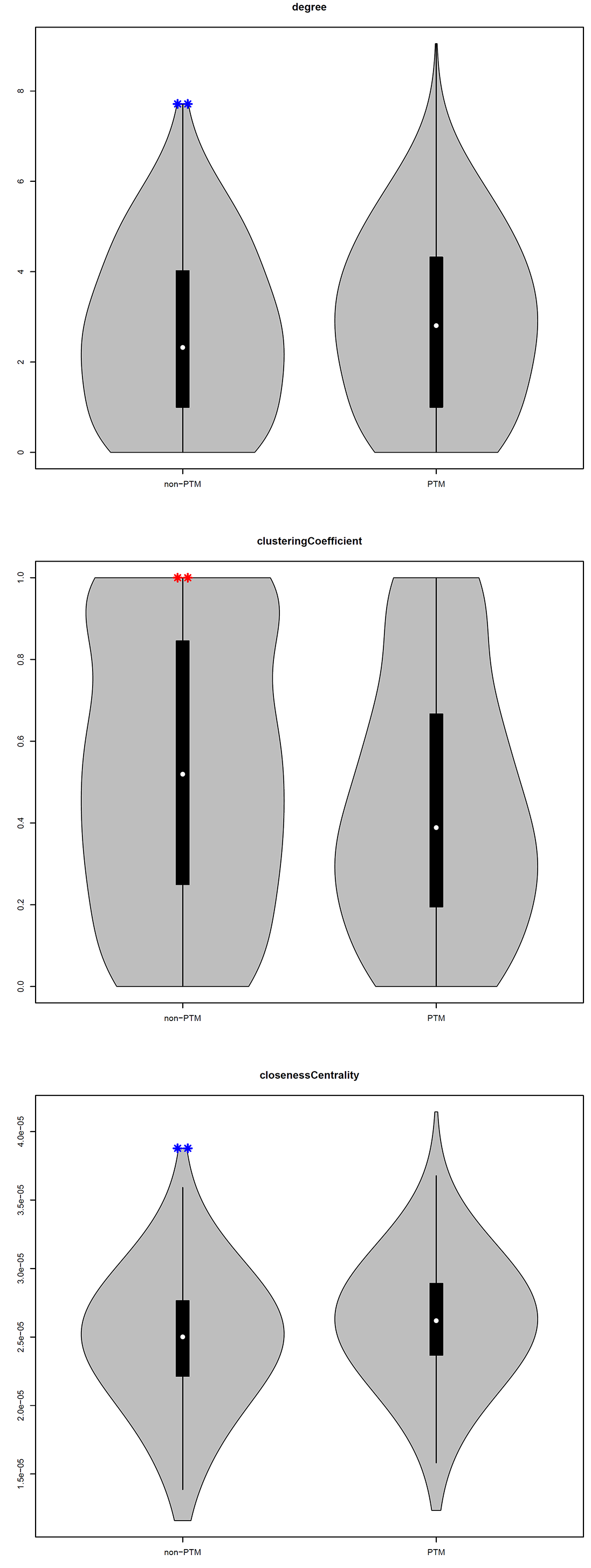

Supplement: S2 Fig — The red (blue) asterisks on the top of violin plot represents the corresponding non-PTM group has a significantly higher (lower) median value compared to the non-PTM group (*: p-value 0.05, **: p-value 0.01) according to a Mann-Whitney test. (TIF) [file pcbi.1004049.s002.tif]

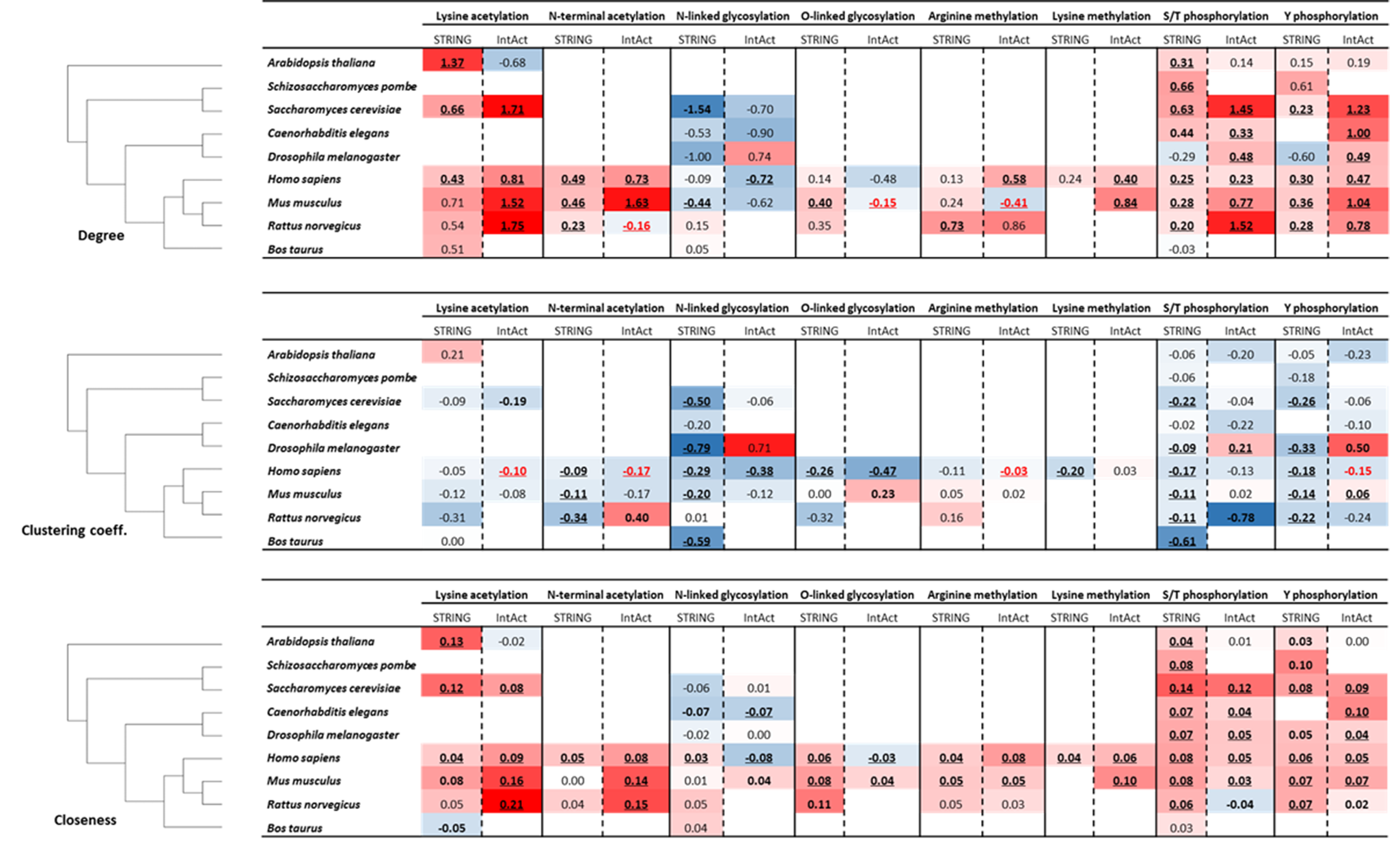

Supplement: S3 Fig — The species are ordered according to their phylogenetic relationships as shown on the left. For every PTM-type, the log-2 of fold difference value for the degree/clustering coefficient/closeness centrality value relative to the respective value associated with proteins not carrying this particular PTM-type are given for PINs based on STRING and IntAct, respectively. Color scale indicates increased (red) or decreased (blue) values in the PTM-set relative to the non-PTM-set with symmetric color intervals (i.e. full color saturation based on the maximal absolute increase or decrease fold difference observed across all values in the table.) Bold-font (underlined) fold-changes indicate significant fold-changes at p<0.05 (p<0.01) by Mann-Whitney test with FDR correction, the values in red or blue text represent significantly higher or lower network properties which are inconsistent with the background color. (TIF) [file pcbi.1004049.s003.tif]

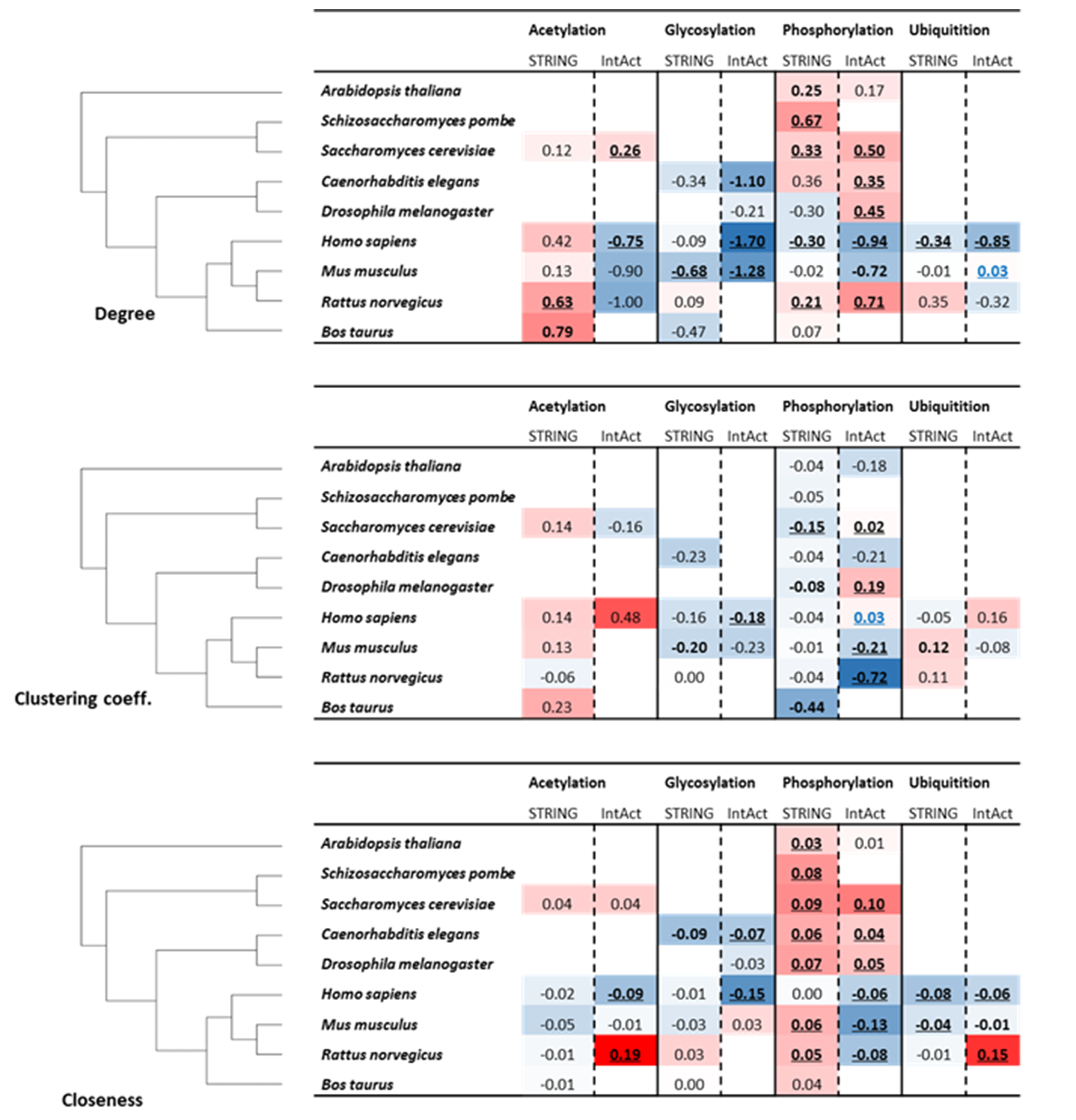

Supplement: S4 Fig — Protein sets were selected to contain one PTM-type only (one-PTM-type-only dataset). The species are ordered according to their phylogenetic relationships as shown on the left. For every PTM-type, the log-2 of fold difference value for the degree/clustering coefficient/closeness centrality value relative to the respective value associated with proteins not carrying this particular PTM-type are given for PINs based on STRING and IntAct, respectively. Color scale indicates increased (red) or decreased (blue) values in the PTM-set relative to the non-PTM-set with symmetric color intervals (i.e. full color saturation based on the maximal absolute increase or decrease fold difference observed across all values in the table.) Bold-font (underlined) fold-changes indicate significant fold-changes at p<0.05 (p<0.01) by Mann-Whitney test with FDR correction, the values in red or blue text represent significantly higher or lower network properties, which are inconsistent with the background color based on mean (not median) values. (TIF) [file pcbi.1004049.s004.tif]

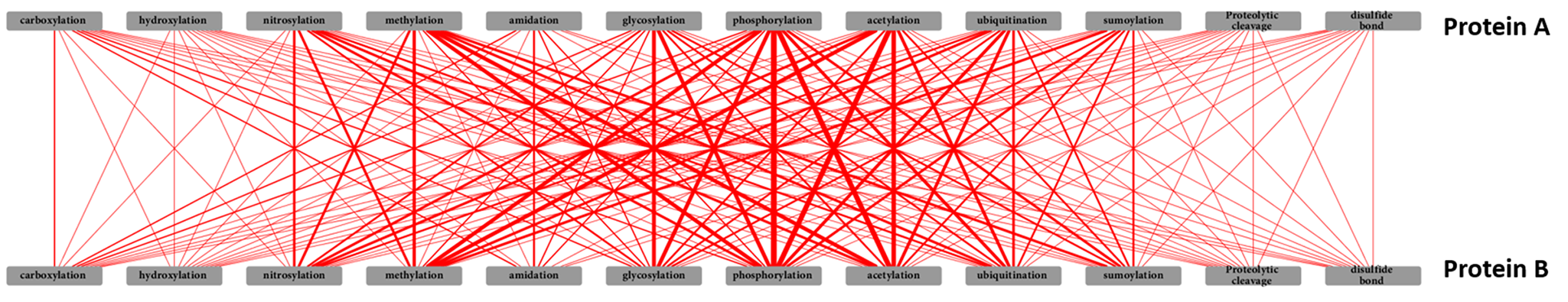

Supplement: S5 Fig — Increased frequencies of protein-protein interactions (designated as protein A and B, respectively) carrying the respective PTM-types relative to expectation. Line width is proportional to the number of species, which exhibit significant interactions of PTM-types carried by interacting proteins. The contingency table for the Fisher exact test contained the respective counts for number of proteins associated with a particular PTM-pair versus all alternative pairings and whether they have been reported to interact or not with applied FDR-corrected p-value threshold of <0.01. (TIF) [file pcbi.1004049.s005.tif]

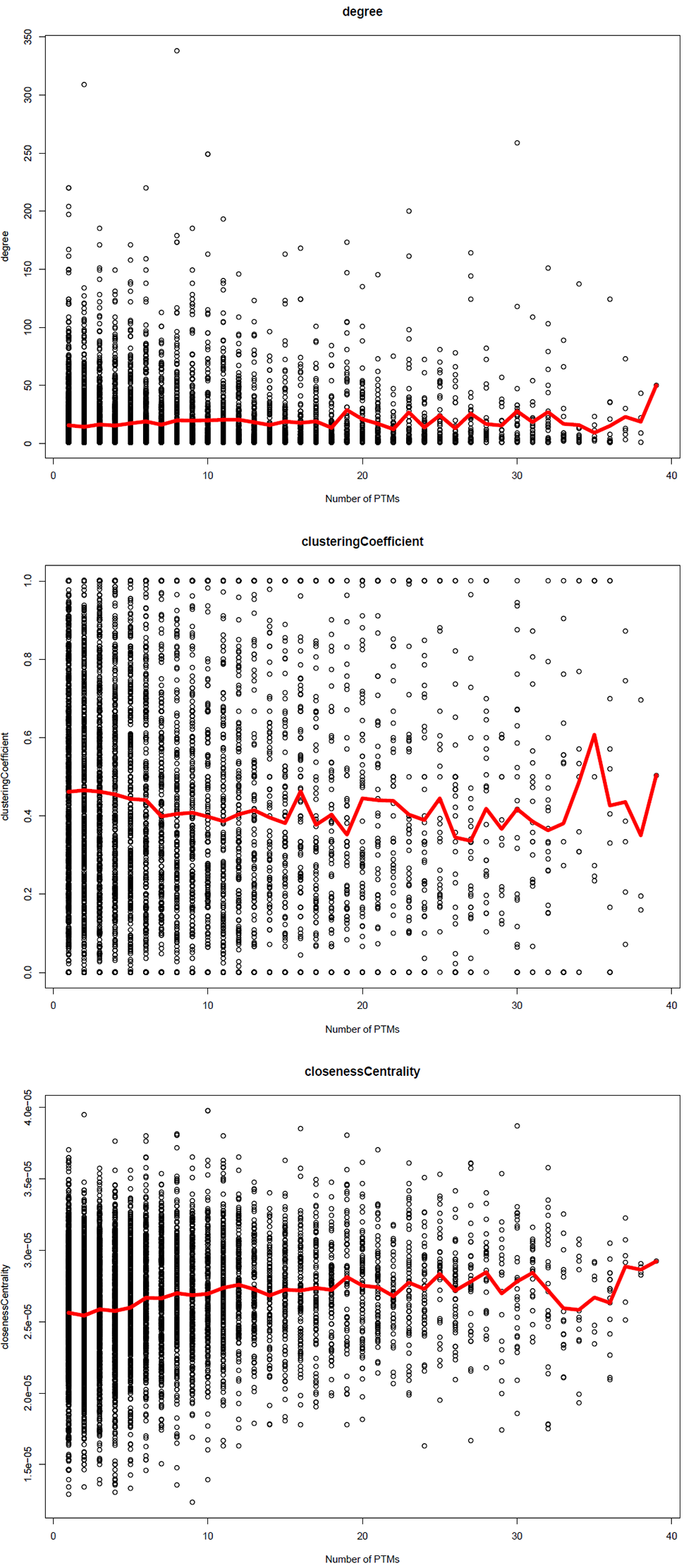

Supplement: S6 Fig — The red line connects the mean network property values of proteins associated with different numbers of PTMs. Associated Pearson linear correlation coefficients, r, (and p-values) were: degree r = 0.056 (1.46E-06), clustering coefficient: r = -0.068 (6.27E-08), closeness centrality: r = 0.164 (0.00). (TIF) [file pcbi.1004049.s006.tif]
