# Supplementary material for: The Roles of Post-translational Modifications in the Context of Protein Interaction Networks
Source: PLoS Comput Biol. 2015 Feb 18;11(2):e1004049. doi: 10.1371/journal.pcbi.1004049 (PMC4333291; doi:10.1371/journal.pcbi.1004049)
Supplement: S1 Table — (DOCX) [file pcbi.1004049.s007.docx]

**Table S1**. Frequency table of proteins associated with the selected PTM-types and species after excluding proteins with more than one PTM-type (one-PTM-type-only dataset); i.e. every protein was annotated to harbor one PTM-type only.

| **NCBI**  **taxonomy ID** | **Species Name** | **acetylation** | **amidation** | **carboxylation** | **disulfide bond** | **glycosylation** | **hydroxylation** | **methylation** | **nitrosylation** | **phosphorylation** | **proteolytic cleavage** | **sumoylation** | **ubiquitination** |
| --- | --- | --- | --- | --- | --- | --- | --- | --- | --- | --- | --- | --- | --- |
| 10090 | *Mus musculus* | 162 | 5 | 2 | 0 | 626 | 0 | 96 | 92 | 4446 | 0 | 6 | 1118 |
| 10116 | *Rattus norvegicus* | 99 | 15 | 5 | 0 | 68 | 4 | 66 | 21 | 2907 | 0 | 5 | 182 |
| 9913 | *Bos taurus* | 66 | 11 | 16 | 0 | 73 | 2 | 6 | 2 | 128 | 0 | 1 | 4 |
| 9606 | *Homo sapiens* | 336 | 7 | 31 | 20 | 426 | 3 | 73 | 9 | 6009 | 53 | 5 | 652 |
| 7227 | *Drosophila melanogaster* | 3 | 12 | 0 | 0 | 53 | 0 | 1 | 0 | 2378 | 0 | 0 | 2 |
| 6239 | *Caenorhabditis elegans* | 5 | 12 | 0 | 0 | 179 | 0 | 0 | 0 | 2093 | 0 | 0 | 0 |
| 3702 | *Arabidopsis thaliana* | 20 | 0 | 1 | 0 | 23 | 0 | 9 | 15 | 9039 | 0 | 3 | 25 |
| 4932 | *Saccharomyces cerevisiae* | 94 | 0 | 0 | 0 | 20 | 0 | 3 | 1 | 2667 | 0 | 0 | 23 |
| 4896 | *Schizosaccharomyces pombe* | 1 | 0 | 0 | 0 | 2 | 0 | 4 | 0 | 1060 | 0 | 0 | 6 |

Listed are the numbers of proteins with the corresponding PTM type in the respective species.
