# Supplementary material for: The Roles of Post-translational Modifications in the Context of Protein Interaction Networks
Source: PLoS Comput Biol. 2015 Feb 18;11(2):e1004049. doi: 10.1371/journal.pcbi.1004049 (PMC4333291; doi:10.1371/journal.pcbi.1004049)
Supplement: S2 Table — (DOCX) [file pcbi.1004049.s008.docx]

**Table S2**. Frequency table of proteins associated with the selected PTM-subtypes and species (PTM-subtypes dataset).

| **NCBI**  **taxonomy ID** | **Species Name** | **N-terminal acetylation** | **Lysine acetylation** | **N-linked glycosylation** | **O-linked glycosylation** | **Arginine methylation** | **Lysine methylation** | **S/T phosphorylation** | **Y phosphorylation** |
| --- | --- | --- | --- | --- | --- | --- | --- | --- | --- |
| 10090 | *Mus musculus* | 1633 | 116 | 1502 | 331 | 825 | 47 | 9219 | 3088 |
| 10116 | *Rattus norvegicus* | 202 | 115 | 110 | 56 | 166 | 35 | 2751 | 1450 |
| 9913 | *Bos taurus* | 17 | 90 | 84 | 36 | 5 | 5 | 158 | 32 |
| 9606 | *Homo sapiens* | 3699 | 1874 | 1378 | 656 | 650 | 488 | 13004 | 7619 |
| 7227 | *Drosophila melanogaster* | 5 | 5 | 80 | 3 | 3 | 3 | 2360 | 321 |
| 6239 | *Caenorhabditis elegans* | 4 | 13 | 206 | 9 | 0 | 2 | 2120 | 102 |
| 3702 | *Arabidopsis thaliana* | 9 | 77 | 41 | 9 | 0 | 14 | 8744 | 1747 |
| 4932 | *Saccharomyces cerevisiae* | 13 | 482 | 52 | 5 | 4 | 11 | 3155 | 511 |
| 4896 | *Schizosaccharo-myces pombe* | 1 | 1 | 11 | 0 | 1 | 4 | 1063 | 58 |
