# Supplementary material for: The Roles of Post-translational Modifications in the Context of Protein Interaction Networks
Source: PLoS Comput Biol. 2015 Feb 18;11(2):e1004049. doi: 10.1371/journal.pcbi.1004049 (PMC4333291; doi:10.1371/journal.pcbi.1004049)
Supplement: S3 Table — (DOCX) [file pcbi.1004049.s009.docx]

**Table S3.** PIN size (number of proteins and interactions) for the nine selected species before excluding the components of size less than 100.

| **NCBI taxonomy**  **ID** | **Species Name** | **STRING** | | **IntAct** | | **Common** | |
| --- | --- | --- | --- | --- | --- | --- | --- |
|  |  | **Proteins** | **Interactions** | **Proteins** | **Interactions** | **Proteins** | **Interactions** |
| 10090 | *Mus musculus* | 7140 | 56313 | 6114 | 14722 | 1149 | 1149 |
| 10116 | *Rattus*  *norvegicus* | 3966 | 19823 | 1445 | 1951 | 254 | 199 |
| 9913 | *Bos taurus* | 3317 | 16498 | 236 | 289 | 151 | 149 |
| 9606 | *Homo sapiens* | 9352 | 71433 | 10650 | 57956 | 3658 | 7596 |
| 7227 | *Drosophila*  *melanogaster* | 4871 | 50684 | 7390 | 24439 | 1123 | 1200 |
| 6239 | *Caenorhabditis*  *elegans* | 4656 | 32870 | 3747 | 7863 | 852 | 809 |
| 3702 | *Arabidopsis*  *thaliana* | 7339 | 36012 | 3692 | 8669 | 1340 | 2304 |
| 4932 | *Saccharomyces cerevisiae* | 4900 | 63279 | 5496 | 68893 | 3117 | 11731 |
| 4896 | *Schizosaccharomyces pombe* | 2981 | 22450 | 0 | 0 | 0 | 0 |

High confidence PINs extracted from STRING are listed in the “STRING” column, the “IntAct” column contains the PIN sizes extracted from IntAct. The “Common” column indicates the number of common proteins and interactions in STRING and IntAct. Only those proteins with available UniProt ID were considered.
